# The Role of Trunk Training for Physical Fitness and Sport-Specific Performance. Protocol for a Meta-Analysis

**DOI:** 10.3389/fspor.2021.625098

**Published:** 2021-06-10

**Authors:** Atle Hole Saeterbakken, Vidar Andersen, David George Behm, Kristoffer Toldnes Cumming, Olaf Prieske, Tom Erik Jorung Solstad, Matthew Shaw, Nicolay Stien

**Affiliations:** ^1^Faculty of Education, Arts and Sports, Western Norway University of Applied Sciences, Sogndal, Norway; ^2^School of Human Kinetics and Recreation, Memorial University of Newfoundland, St. John's, NL, Canada; ^3^Faculty of Health and Welfare Sciences, Østfold University College, Fredrikstad, Norway; ^4^Division of Exercise and Movement, University of Applied Sciences for Sports and Management Potsdam, Potsdam, Germany

**Keywords:** sport performance, trunk, trunk stability, trunk strength, athletic performance

## Abstract

The trunk (core) muscles are involved in daily functions (i. e., stabilizing the body in everyday tasks) and force generation of the limbs during athletic tasks such as kicking, throwing, or running. Even though trunk training is a popular means for improving physical fitness and athletic performance, the direct relationship of improved trunk function (i.e., stability, strength, or endurance), fitness and sport-specific performance is not conclusive. The aim of this proposed review is to evaluate the effects of trunk training on physical fitness and sport-specific performance, and to examine potential subject-related (e.g., age, sex) and trunk training-related moderator variables (e.g., training period, training frequency) for performance changes. We will conduct a systematic literature search in Web of Science, MEDLINE (via EBSCO) and SportDiscus. Relevant papers will be screened independently by two reviewers in two stages: (1) title and abstracts and (2) the full text of the remaining papers. A third reviewer will resolve possible disagreements. Data extraction and risk of bias of the included studies will be performed in addition to the PEDro scoring to judge the quality of the studies. A meta-analysis will be conducted to determine the efficacy of trunk training to increase physical fitness and sport-specific performance measures. In addition, subgroup univariate analyses were computed for subject-related (i.e., age, sex, performance level) and training-related moderator variables (i.e., training period, training frequency, training sessions, session duration). The results of this proposed systematic review and meta-analysis will assess the effects of trunk training on physical fitness and sport-specific and identify which subject-related and training-related moderate variables of trunk training modality might be beneficial for performance gains. This knowledge has potential importance for athletes and coaches in sports.

## Introduction

The trunk of the body includes the spine, hip and pelvis, whereas the trunk muscles can be defined as muscles supporting the lumbopelvic-hip complex (Akuthota and Nadler, 2004; Borghuis et al., 2008). The muscles and joints of the lumbopelvic-hip complex are involved in daily functions (i.e., stabilizing the body in everyday tasks) and force transmission of the limbs during athletic tasks such as kicking, throwing, or running (Kibler et al., 2006; Saeterbakken et al., 2011). The trunk muscles provide proximal stability for distal mobility, which involves more than muscles directly attached to the spine and pelvis (local and global stabilizers) (Bergmark, 1989; Panjabi, 1992). For example, most of the prime movers of the upper- and lower extremities (glutes, hamstrings, latissimus dorsi, pectoralis major) are attached to the trunk. Therefore, the trunk and its central position in the body, transfer and control force and motion in an integrated kinetic chain and is crucial in every athletic function (Kibler et al., 2006; Behm et al., 2010).

Although trunk training is popular for targeting athletic development and improving performance, the direct relationship between improved trunk function (i.e., stability, strength or endurance), physical fitness and sport-specific is not conclusive (Kibler et al., 2006; Willardson, 2007; Hibbs et al., 2008; Reed et al., 2012). Despite several studies reporting positive results among recreationally active participants (Hibbs et al., 2008; Reed et al., 2012), generalizing these results to competitive athletes is often difficult. Furthermore, athletes rarely perform training programs only involving the trunk, but use high-intensity ground-based multi-joint exercises (e.g., squats, dead lifts, Olympic lifts) involving the trunk muscles (Hamlyn et al., 2007; Nuzzo et al., 2008; Saeterbakken et al., 2019). Therefore, it could be difficult to isolate the effects of training of the trunk.

Still, and to the authors' best knowledge, only two previous systematic reviews have focused on the effects of trunk training on physical fitness and sport-specific performance (Reed et al., 2012; Prieske et al., 2016). However, and importantly, the review by Reed et al. (2012) did not distinguish between the different trunk training approaches, the literature search ended in June 2011 and no meta-analysis was conducted. Furthermore, the review by Prieske et al. (2016) focused on the association and physical performance (i.e., trunk—and muscle strength) among trained individuals in addition to athletic performance in trunk strengthening interventions. The authors displayed a small-sized relationship between measures of trunk muscle strength and physical fitness/sport-specific performance outcomes. Additionally, small-to-moderate-sized effects of trunk training on physical fitness and sport-specific performance were observed. Still, Prieske et al. (2016) did not include sub-group analysis of potential moderators. Therefore, it is not known how different trunk training related variables (e.g., length of the training period, training session frequency, session duration) and subject related variables (e.g., age, performance level, sex) affects physical fitness (e.g., jumping performance) or sport-specific performance outcomes (e.g., throwing velocity, drive distance or swim time) in competitive athletes. These factors are important modifying factors of strength training and performance gains (Moran et al., 2017, 2018; Chaabene et al., 2020). Furthermore, both healthy trained individuals and athletes were included in the analysis that may have contributed to the heterogeneity among the included studies (Prieske et al., 2016).

Since the pioneer work by Prieske et al. (2016), the scientific literature on trunk training has grown immensely. Thus, a systematic update on the effects of trunk training on physical fitness and athletic function in athletes appears to be timely and imperative. Original papers, systematic reviews, and meta-analysis are called upon to expand the scientific literature and strengthening the decision-making process among coaches and athletes. The research questions to the proposed meta-analysis are:

1) What is the effect of trunk training programs toward physical fitness and sports-specific performance measures?
2) What is the effect of trunk training programs toward trunk related outcomes like strength and maintain a position (i.e., prone bridge)?
3) Do subject related variables (e.g., sex or performance level) affect sport-specific performance and physical fitness performance differently?
4) Do the different trunk training approaches (length of the training period, weekly training sessions and duration of each session) affect sport-specific performance and sports-related outcomes differently?
5) Do young athletes (>10–13 years) benefit more from trunk training than adolescents (>13 to ≤18 years) or adults (>18 years)?

## Methods and Analyses

The international prospective register of systematic reviews (PROSPERO) does not accept sport performance outcomes. Therefore, and according to current recommendations, the aim of this paper is to describe the protocol for a systematic review according to PRISMA-P 2015 guidelines for systematic reviews (Supplementary Material 1) and meta-analyses (Moher et al., 2015; Shamseer et al., 2015). Studies will be selected according to the criteria described.

### Study Design

We will include quantitative studies as cohort, longitudinal design, and randomized controlled trials. Pilot-studies, methodological studies, and literature reviews will be excluded. Hence, a specific trunk training intervention aiming to improve trunk strength, endurance or stability must be conducted to be included. General resistance training programs including trunk exercises or exercises including the trunk in the execution (i.e., squat or deadlift), will not be included.

### Subjects

There will be no restrictions of sex or sport. Studies involving participants under 10 years and above 60 years will be excluded. Furthermore, the participants must compete in a defined sport and with a sport-specific performance outcome (i.e., throwing- and kicking velocity, time-trials in running or cycling) or physical fitness outcomes (i.e., muscular power, acceleration, jump performance, linear sprint). Trunk training programs including non-competing sport athletes (i.e., active, students), patient groups or rehabilitation groups will be excluded. Studies not reporting sports performance measurements will also be excluded.

### Interventions and Comparators

Studies will have had to perform trunk training in addition to regular sport training at least once a week, over a minimum period of 5 weeks. We will subdivide the trunk training programs into trunk stability, trunk strength and trunk endurance. Studies which have combined the three different approaches, will be quantified as one of the approaches based on the which training modality is used most frequently within a training program. For example, a study will be considered a trunk stability if participants performed trunk stability three times per week and trunk strength once per week. Studies will be excluded if it is difficult to define the training approaches as one of the three strategies. Studies will be excluded if they do not measure sports performance variables and only trunk training outcomes (i.e., endurance, strength, or stability of the trunk) or if trunk training is a part of a rehabilitation program/intervention among injured athletes (i.e., not competing in sport). Finally, a comparator group (passive, active or alternative training) needs to be included. Studies with a two-armed trunk muscle training intervention was excluded where none of the intervention could serve as a control condition.

### Outcomes

The primary outcomes are sport-specific performance measurements such as time trials (i.e., running, swimming, rowing, skiing, or cycling), velocity (i.e., kicking, throwing, or swinging), explosive and strength characteristics (i.e., jump height, acceleration, sprinting, mechanical power, or muscle strength) and other discrete quantitative measures directly related to physical fitness. Other sport-specific performance measures (i.e., tactical, mental, social) will be excluded. The secondary outcomes are trunk-related measurements such as time to maintain position or numbers of repetitions to fatigue (trunk endurance tests), trunk stability tests measured with center of pressure and trunk strength tests (dynamic and isometric) isolating the trunk muscles. Furthermore, both subject-related variables and training related variables will be extracted as moderators. Subject related variables include sex, age, and performance level. According to previous definitions (Chaabene et al., 2020; Thiele et al., 2020), age will be categorized as children (>10–13 years), adolescents (>13 to ≤18 years) and adults (>18 years), and performance level as recreational/sub-elite or elite athletes Training-related variables include training period length, weekly training frequency, total number of training sessions, and duration of each training session (Chaabene et al., 2020).

### Period and Settings

All articles that fit the inclusion criteria will be included, regardless of date of publication. The only restrictions to a setting are that a trunk training intervention has to be conducted using competing and healthy sport athletes and are available in full text.

### Language

Articles published in English and Scandinavian language (i.e., Norwegian, Swedish and Danish) will be included. Articles in any other language will be excluded.

### Search Details

A systematic search strategy will be developed with a special librarian in these databases: Web of Science, MEDLINE (via EBSCO) and SportDiscus. The trunk stability on athletic performance review by Reed et al. (2012), contains a literature search through 2012. Their search strategy will be used, but we will include other terms for the trunk (i.e., trunk, lumbopelvic) and sport performance measurements (i.e., velocity, time, height). In addition, we will manually inspect the reference lists of included studies and previous reviews and position stands (Willardson, 2007; Hibbs et al., 2008; Behm et al., 2010; Reed et al., 2012; Prieske et al., 2016) for relevant studies. Finally, to ensure all studies and/or unpublished material are included, two experts in the field will be asked to provide a list of five key articles within the area of trunk training. This will be done to examine if any published material unknown for the authors which meet the inclusion criteria, should be considered for inclusion.

### Search Strategy

A librarian with expertise in systematic review search strategies will help develop the search strategy based on the following domains: athletic/sport performance and trunk training intervention studies. A draft of the search strategy and PICO form (Problem, Intervention, Comparison, and Outcome) are added as supplemented file and available online (Supplementary Material 2).

The final search, and search history will be saved in respective databases. The final search will be performed again ahead of publishing to make sure the review covers all published material.

### Data Extraction

The data extraction process will be completed in accordance with the Cochrane Handbook (www.handbook.cochrane.org). All identified records will be imported to EndNoteX9 with PDF attached. The field exported from the database will according to the Cochrane Handbook (Higgins and Green, 2008) contain abstract, accession number/unique identifier, affiliation/address, article identifier/ digital object identifier (DOI), clinical trial number, index terms/thesaurus term/keywords, language and comments/corrections, errata, retractions, and updates. Search results will be merged into one valid library and duplicates of the same report will be deleted.

### Selection Process

The study selection process will follow a three-step process.

Two researchers (AHS and NS) will review the titles of search results independently. In case of doubt, the record will be included in the abstract review phase.

The same two researchers (AHS and NS) will independently and blinded examine abstracts to assess for eligibility of inclusion. Records will be coded as “yes” or “no.” In case of doubt, the record will be included in the review of full-text phase.

Full text of all included articles will be retrieved with eligibility criteria by two researchers (AHS and NS). Records will be coded as “yes,” “no,” or “maybe.” In case of disagreement, this will be solved by a third author (VA). There will be a consensus for all included studies and a record will be kept of reasons for excluding studies.

### Data Extraction

We will extract the following data from the included studies: (1) general information such as authors, year of publication, sport and study design. (2) Participants' characteristics such as sex, age, height, mass, sport experience and competitive level. (3) Study characteristics as sample size, training duration, training volume and trunk training method. (4) Outcomes such as sport-specific performance outcomes (i.e., throwing or kicking velocity), sport-related outcomes (i.e., muscular power, linear sprint, change of direction, and agility) and trunk outcomes (i.e., stability, strength or endurance) at baseline and end of intervention.

### Risk of Bias (Quality) Assessment

The quality and the risk of bias in the included studies will systematically be assessed by following the Cochrane handbook for systematic reviews of interventions (Higgins and Green, 2008; Higgins et al., 2011). Selection bias, performance bias, detection bias, attrition bias and reporting bias will be reported. Two reviewers (AHS and NS) will independently rate the bias in each study as high, low or unclear. If disagreements, a third reviewer (VA) will be included in the discussion to come to a consensus.

### Quality of Each Included Paper

The same reviewers (AHS and NS) will independently strunk the methodological quality of each paper using the 11-point Physical Therapy Evidence Database (PEDro) scale (Maher et al., 2003). Strunks will be assigned based on the fulfillment of the 11 criteria design to assess internal and external variability. The PEDro scale range from 0 to 10. Studies ranging from 6 to 7 will be of “excellent quality,” 5 being “good quality, 4 being “moderate quality,” and 0–3 being “poor quality” as previous exercise interventions reviewers (Kummel et al., 2016; Grgic et al., 2017). We will try to include studies with a trunk of ≥5 from the PEDro Scale (0–10) (Maher et al., 2003), but the score itself will not be the criteria for inclusion or exclusion. Points will only be awarded when a study clearly meets the criteria on a literal reading. If there is a disagreement of the rating score between reviewers, a third assessor (VA) will be obtained to achieve a consensus.

### Strategy for Data Synthesis

Data synthesis is developed in accordance with guidelines for Systematic reviews in health care (Egger et al., 2008). Results from all included studies will be tabulated. Possible sources of heterogeneity will be explored. Both forest plot, and funnel plots will be examined. In addition, a sensitivity analysis will be performed. The main analyses will be restricted to studies with low risk of bias.

A meta-analysis will be conducted to systematically aggregate the effects of trunk training on physical fitness and sport-specific performance measures. In addition, sub-group univariate analyses of subject-related moderate variables (i.e., sex, performance level, age) and training-related moderate variables (length of the training period, weekly training frequency and total number of training sessions, duration of each session) are planned.

The meta-analysis will be conducted using Comprehensive meta-analysis (https://www.meta-analysis.com) to determine the efficacy of trunk training for increasing sport performance measurement. Data will be pooled in three subgroups trunk stability, trunk strength and trunk endurance. Outcomes will be analyzed as continuous outcomes using random effects model to calculate a standardized mean difference with 95% confidence intervals. A *p*-value < 0.05 will indicate a statistical significance for an overall effect. In case of missing data, we will contact the authors to obtain the missing data. In addition, the authors will also be contacted if only effects size estimates are presented.

The analysis will include between-study heterogeneity (*I*^2^) and chi-square statistics (*X*^2^) to examine whether the proportion of effects were caused by heterogeneity or chance (Liberati et al., 2009). *I*^2^ outcomes of 25, 50, and 75% correspond to low, moderate, and high heterogeneity (Higgins et al., 2003). In addition, chi-square statistic (*X*^2^) will be included to determine whether the results of the analysis were due to chance.

### Quality of the Evidence

The quality of the evidence of the main outcome (sport performance) will be judged using the Grading of Recommendations Assessment, Development of Evaluation (GRADE) approach. Risk of bias, consistency (i.e., variability or heterogeneity of the results), precision and risk of publication bias, and consistency (i.e., indirect or direct comparison of training intervention, differences in sport athletes). The quality of the evidence will be judged as very low, low, moderate or high based on whether further studies is very uncertain, very likely, likely or very unlikely to change confidence in the estimated effect (Guyatt et al., 2008).

## Discussion

The importance of a functional trunk to produce local stability and distal mobility through optimizing production, transfer, and control of force between the feet (i.e., running), arms (i.e., swimming) or from lower- to upper-body (i.e., golf swing) is being increasingly recognized. Athletes and coaches aiming to improve sport-specific have included trunk training programs, but little is still known about the direct effects of improving trunk function on sport-specific (e.g., running, kicking, throwing). In a systematic meta-analytic review, we expect to identify whether trunk training improves sport-specific and if different subject related or training related variables will be more effective than other programs.

To the authors‘ best knowledge, only one previous systematic review (Reed et al., 2012) and one meta-analysis (Prieske et al., 2016) focusing on trunk training and athletic performance have been conducted. Of the 24 studies included in the review by Reed et al. (2012), a diverse range of populations and intervention styles were included. Further, Prieske et al. (2016) included trained athletes and did not examine potential moderators in sub-group analysis. In the present study, we will only include active competing athletes and try to examine potential moderator variables to expand the understanding of mechanisms and attributors in trunk training. To the authors best knowledge, this has not been done yet and will be of significant for coaches and athletes. Potential, this may fill the gap of knowledge of the different trunk training approaches and performance and further provide a deeper insight in trunk training, physical fitness and sport-specific performance including the scientific literature from the latest decade.

The search strategy will be developed with help from an experienced research librarian. We have included a broad search term to discover all appropriated articles in addition to invite two experts in the field to add five key-papers. Importantly, broad search terms will be used, but the inclusion criteria are limited to active competitive athletes and to sport-related outcomes. Therefore, this detailed protocol paper represents a major strength of the systematic review. To the authors best knowledge, the first meta-analyses (Prieske et al., 2016) and systematic review (Reed et al., 2012) examining trunk training and athletic performance and had no conclusive conclusion on the effects of trunk training toward athletic function (Reed et al., 2012). Due to limited numbers of studies, the review included few papers with highly trained competitive athletes, and instead, had to include papers with a population of recreationally active students or adults (Reed et al., 2012). However, the scientific literature on trunk training has grown massively in the last decade since the review by Reed et al. (2012) were conducted and the need for a new summary of the current scientific knowledge is needed. Therefore, we believe it is time to examine competitive athletes.

In this review, we plan to conduct a risk of bias assessment and only include studies with low risk of bias. In addition, we plan to score the included articles using the PEDro scale (Maher et al., 2003) and report the scores. In the previously mentioned review (Reed et al., 2012) and meta-analysis (Prieske et al., 2016), the PEDro scores ranged from 2 to 7 using the 10 points scale. If possible, we hope to include articles with a minimum score of five. However, there is an inherent risk and problem to lose the precision when excluding studies with high or unclear risk of bias or a low PEDro score. The *in-vivo* setting of sport intervention may not be feasible compared to clinical setting even when the researchers are striving to use the best methodological approach possible. Still, reducing the risk of biases are more important than the precision.

A limitation of the present protocol is the inclusion of studies only published in English and Scandinavian languages. This may represent a bias. Still, the effects of language bias might be diminished by an increasing number of studies published in English the last decades (Higgins and Green, 2008). Furthermore, trunk training is almost never the sole training program being performed but is included as part of a larger training regime, which may also be a limitation. A position stand suggests that ground-based free weights exercises can provide a stress on the trunk muscles (Behm et al., 2010). Ground-based free weights exercises are used to develop strength, power and hypertrophy in athletic training. The isolated trunk training effects may therefore be masked by traditional resistance training programs. Finally, having a trunk training group performing an extra training session (i.e., greater overall training volume per week) compared to a control group, may represent a limitation.

## Ethics Statement

Ethical review and approval was not required for the study on human participants in accordance with the local legislation and institutional requirements. Written informed consent for participation was not required for this study in accordance with the national legislation and the institutional requirements.

## Author Contributions

AHS conceived the initial idea and wrote the first draft. VA, KTC, DGB, OP, NS, MS, and TS contributed to developing the idea, data extraction strategy, risk of bias assessment, PEDro score, and selection criteria. OP had the idea of the sub-group analyses and the distribution of the different outcomes. All authors critically reviewed the manuscript and approved the final version.

## Conflict of Interest

The authors declare that the research was conducted in the absence of any commercial or financial relationships that could be construed as a potential conflict of interest.

## Supplementary Material

Supplementary Material 1 PRISMA-P 2015 checklist.

Supplementary Material 2 Search strategy and PICO form.
