# Supplementary material for: The Role of Trunk Training for Physical Fitness and Sport-Specific Performance. Protocol for a Meta-Analysis
Source: Front Sports Act Living. 2021 Jun 10;3:625098. doi: 10.3389/fspor.2021.625098 (PMC8222678; doi:10.3389/fspor.2021.625098)
Supplement: Supplementary Material 1 — PRISMA-P 2015 checklist. [file Data_Sheet_1.docx]

| **Supplementing file 2** | | | | |
| --- | --- | --- | --- | --- |
| **PICO** | **Search term** | **MEDLINE**  **(**via EBSCO) | **Sportsdisc** | **Web of Science** |
| **P**  **Patient/**  **problem** | 1. Athletes* 2. Players* 3. #1 OR #2 | 1.  2.  3. | 1.  2.  3. | 1.  2.  3. |
| **I**  **Intervention** | 1. “core* training*” 2. “core* strength*” 3. “core* endurance*” 4. “core* stability*” 5. “trunk* training*” 6. “trunk* strength*” 7. “trunk* endurance*” 8. “trunk* stability*” 9. #4 OR #5 OR #6 OR #7 OR #8 OR #9 OR #10 OR #11 | 4.  5.  6.  7.  8.  9.  10.  11.  12. | 4.  5.  6.  7.  8.  9.  10.  11.  12. | 4.  5.  6.  7.  8.  9.  10.  11.  12. |
| **C**  **Comparison** | (sport athletes) |  |  |  |
| **O**  **Outcomes** | 1. “Performance” 2. “velocity” 3. “speed” 4. “height” 5. “distance” 6. “time” 7. #13 OR #14 OR #15 OR #16 OR #17 OR #18 | 13.  14.  15.  16.  17.  18.  19. | 13.  14.  15.  16.  17.  18.  19. | 13.  14.  15.  16.  17.  18.  19. |
| **Study design** | 1. “Training* intervention” 2. “Training* period*” 3. #20 OR #21 | 20.  21.  22. | 20.  21.  22. | 20.  21.  22. |
| Combined search | 1. #3 AND #12 AND #19 AND #22 | 23. | 23. | 23. |
| Limitation | English, original paper, peer-review |  | | |
|  |  |  | | |
