# Supplementary material for: The Role of Trunk Training for Physical Fitness and Sport-Specific Performance. Protocol for a Meta-Analysis
Source: Front Sports Act Living. 2021 Jun 10;3:625098. doi: 10.3389/fspor.2021.625098 (PMC8222678; doi:10.3389/fspor.2021.625098)
Supplement: Supplementary Material 2 — Search strategy and PICO form. [file Table_1.docx]

| **Supplementing file 1** | | | | |
| --- | --- | --- | --- | --- |
| **PICO** | **Search term** | **MEDLINE**  **(**via EBSCO) | **Sportsdisc** | **Web of Science** |
| **P**  **Patient/**  **problem** | 1. Athletes* 2. Players* 3. #1 OR #2 | 1.  2.  3. | 1.  2.  3. | 1.  2.  3. |
| **I**  **Intervention** | 1. “core* training*” 2. “core* strength*” 3. “core* endurance*” 4. “core* stability*” 5. #4 OR #5 OR #6 OR #7 | 4.  5.  6.  7.  8. | 4.  5.  6.  7.  8. | 4.  5.  6.  7.  8. |
| **C**  **Comparison** | (sport athletes) |  |  |  |
| **O**  **Outcomes** | 1. “Performance” 2. “velocity” 3. “speed” 4. “height” 5. “distance” 6. “time” 7. #9 OR #10 OR #11 OR #12 OR #13 OR #14 | 9.  10.  11.  12.  13.  14.  15. | 9.  10.  11.  12.  13.  14.  15. | 9.  10.  11.  12.  13.  14.  15. |
| **Study design** | 1. “Training* intervention” 2. “Training* period*” 3. #17 OR #18 | 16.  17.  18. | 16.  17.  18. | 16.  17.  18. |
| Combined search | 1. #3 AND #8 AND #15 AND #18 | 19. | 19. | 19. |
| Limitation | English, original paper, peer-review |  | | |
|  |  |  | | |
